# Pharmacogenomics study on cadherin 2 network with regard to HIV infection and methadone treatment outcome

**DOI:** 10.1371/journal.pone.0174647

**Published:** 2017-03-30

**Authors:** Hsiang-Wei Kuo, Chia-Lung Shih, Jieh-Hen Tsung, Sheng-Wen Liu, Shih-Kai Chu, Hsin-Chou Yang, Hsiao-Hui Tsou, Zih-Hsiang Wang, Andrew C. H. Chen, Yu-Li Liu

**Affiliations:** 1 Center for Neuropsychiatric Research, National Health Research Institutes, Miaoli, Taiwan; 2 Institute of Statistical Science, Academia Sinica, Taipei, Taiwan; 3 Division of Biostatistics and Bioinformatics, Institute of Population Health Sciences, National Health Research Institutes, Miaoli, Taiwan; 4 Graduate Institute of Biostatistics, College of Public Health, China Medical University, Taichung, Taiwan; 5 Department of Psychology, National Taiwan University, Taipei, Taiwan; 6 Department of Psychiatry, The Zucker Hillside Hospital, Northwell Health, Glen Oaks, New York, United States of America; 7 The Feinstein Institute for Medical Research, Hofstra Northwell School of Medicine at Hofstra University, Manhasset, New York, United States of America; 8 Graduate Institute of Clinical Medical Science, China Medical University, Taichung, Taiwan; South Texas Veterans Health Care System, UNITED STATES

## Abstract

Heroin dependent patients have a high incidence of HIV infection. In contrast to the gene expression method, we developed a systemic correlation analysis method built upon the results of pharmacogenomics study in a methadone maintenance treatment (MMT) cohort consisting of 344 Taiwanese heroin dependent patients. We identified genetic variants and their encoding proteins that may be involved with HIV infection and MMT treatment outcome. Cadherin 2 (*CDH2*) genetic determinants were identified through the genome-wide pharmacogenomic study. We found significant correlations among HIV infection status, plasma levels of CDH2, cytokine IL-7, ADAM10, and the treatment responses to methadone. Two single nucleotide polymorphisms located within *CDH2* gene showed associations with blood pressure and plasma CDH2 concentration. Plasma concentration of CDH2 showed correlations with the level of cytokine IL-7, status of HIV infection, and urine morphine test result. Plasma level of IL-7 was correlated with corrected QT interval (QTc) and gooseflesh skin withdrawal symptom score, while level of ADAM10 was correlated with plasma concentrations of vitamin D metabolite, nicotine metabolite, and *R*-methadone. The results suggest a novel network involving HIV infection and methadone treatment outcome.

## Introduction

Pharmacogenomics study has recently received recognition for its potential application in personalized medicine [1]. One of the methods in pharmacogenomic analysis is to build pathway network and observe gene-gene interaction status contributing to treatment responses. However, this kind of analysis often misses the opportunity to discover novel plasma proteins that may serve as potential biomarkers toward treatment response. In this study, we developed a new method by starting genetic variant association analyses, determining the plasma levels of proteins encoded by the candidate genes, then finally examining the protein-phenotype (treatment responses) correlations in a Taiwanese methadone maintenance treatment (MMT) population. Through this new approach, we were able to identified novel network among the gene encoding proteins involving cardiovascular function, inflammatory factors, and MMT treatment responses.

MMT is one of the standard treatments for heroin dependence [2]. Cardiovascular side effects are one of the major concerns for methadone treatment [3]. The most well known cardiovascular side effect is QT prolongation [4]. Recent studies have revealed a few novel molecules that may be involved with cardiovascular function. Cadherin 2, type 1, *N*-cadherin (neuronal) (CDH2) is a member of the cadherin superfamily [5]. *N*-cadherin is expressed in vascular smooth muscle cells [6] and endothelial cells [7]. It mediates smooth muscle cell migration [8] and cell-cell adhesion [9]. In addition to supporting cell junctions, *N*-cadherin also plays a role in maintaining normal cardiovascular function [10]. For example, a rat study [11] showed that *N*-cadherin antibody can inhibit the arteriolar myogenic reactivity, which regulates blood pressure, suggesting that CDH2 is involved with the mechanisms underlying regulation of blood pressure.

The protein structure of CDH2 is composed of five cadherin repeats in the N-terminal of extracellular domain. This domain contains four Ca^2+^-binding sites and is involved in homotypic protein–protein interaction where the extracellular domain interacts between adjacent cells in an anti-parallel conformation and creates a linear, adhesive "zipper" between cells [12, 13]. The extracellular domains of CDH2 determine the basic localization pattern, whereas the C-terminal, cytoplasmic domains, modulate its effects on the cell adhesion activity, subapical accumulation, and the formation of adherent junction [14]. Soluble CDH2 stimulates migration of endothelial cells in the wound healing process as it promotes angiogenesis [15]. Plasma level of CDH2 may be regulated by matrix metalloproteinase. ADAM metallopeptidase domain 10 (ADAM10) has been reported to cleave the extracellular domain of CDH2 and to regulate its plasma level [16, 17]. ADAM10 is a cell surface protein involving emphysema caused by cigarette smoking [18], and metabolism of vitamin D related to atherogenesis [19]. In this study, we analyzed the plasma level of ADAM10 to explain in part the mechanisms underlying the regulation of plasma CDH2 level.

Inflammatory factors have been reported to influence the progress of heroin dependence [20]. Interleukin-7, IL-7, is a cytokine released from bone marrow stromal cells, hepatocytes and neurons [21]. IL-7 has been reported to be involved with certain heart diseases [22, 23], and skin inflammatory process [24]. In our previous study regarding the correlation between plasma protein levels and MMT treatment response [25], we discovered a novel correlation between IP-10 protein and cardiac response in heroin dependent patients. Built upon the method, we identified a significant correlation between the CDH2 protein network and its metabolic enzyme ADAM10 and cytokine IL-7 through systemic relation analyses in the present study.

## Methods

### Methadone maintenance subjects

The study protocol was approved by the institutional review boards of the National Health Research Institutes (EC0970504)(Zhunan, Taiwan) and the six participating hospitals of Tao-Yuan Mental Hospital, En-Chu-Kong Hospital, Far-Eastern Memorial Hospital, Taipei City Hospital Song-De and Yang-Ming Branches, China Medical University Hospital, and Wei-Gong Memorial Hospital. Written informed consents were obtained from all participants. The project was registered with the National Institutes of Health Clinical Trial database (http://www.clinicaltrial.gov/ct/show/NCT01059747). 360 Taiwanese MMT subjects were recruited. The inclusion criteria were as follows: age of 18 years or above, receipt of MMT for at least three months with regular attendance in the past seven days, and methadone dosage adjustment of no more than 10 mg in the past seven days. Exclusion criteria were co-morbidity with severe mental disorders, such as, organic mental disorders, schizophrenia, or severe cognitive impairment requiring emergent treatment, and pregnancy.

### Clinical assessments

Demographics, clinical characteristics and methadone treatment courses, including the dose of methadone and treatment duration, other medications, treatment adherence over the past one week, were obtained from their medical records. Several interviewer-administered assessments, including Clinical Opioid Withdrawal Scale (COWS) for evaluating the severity of 11 opioid withdrawal symptoms [26], were conducted before the next methadone was administered. Urine morphine screen was performed as a surrogate for treatment outcome. Plasma level of vitamin D metabolite, 25-hydroxyvita- min D, was assayed by EIA kit (Immunodiagnostic Systems Ltd., Fountain Hills, Arizona). Plasma concentrations of nicotine metabolite cotinine [27] and methadone [28] were assayed by the methods reported in our previous reports. The serum samples were examined for anti-HCV antibody, and anti-HIV antibody by electrochemiluminescence immunoassay (ECLIA).

### Electrocardiogram (ECG) and blood pressure measurement

The electrocardiogram (ECG) was performed in each participating hospital according to the regular standard operation procedure (SOP) as previously described [29]. The QT interval, corrected for heart rate according to the Bazett's formula (QTc), was used for subsequent analysis [30]. The QTc change represents the difference between baseline and current QTc intervals for patients with a complete set of baseline and current ECG measurement data. Blood pressure was measured before the next dose of methadone.

### CDH2 and genomewide genotyping

The genomic DNAs were extracted from the buffy coat of 6 ml whole blood lymphocyte pellets using the Puregene^®^ Blood kit C (QIAGEN Sciences; Maryland, USA). All samples were genotyped at the National Center of Genomic Medicine (Taipei, Taiwan) using the Axiom Genome-Wide CHB 1 Array (Affymetrix, Inc., San Diego, CA, USA) with a better genomic coverage of common alleles in the Han Chinese genome. Details of the sample quality controls (sex check, sample call rate, homozygosity, cryptic relatedness, and divergent ancestry) and SNP quality controls (SNP call rate, minor allele frequency, and Hardy-Weinberg equilibrium test) were reported in our previous manuscript [31]. Confirmation genotyping was further verified with probe C_29000295_10 of Applied Biosystems TaqMan SNP genotyping assay then analyzed by the StepOnePlus Real-time PCR System (Applied Biosystems, Foster City, USA) according to the manufacture’s protocol on *CDH2* SNP rs8094439.

### Plasma CDH2 and ADAM10 ELISA assay

Plasma levels of CDH2 and ADAM10 were measured by the enzyme-linked immuno-sorbent assay (ELISA) kits according to the manufacture instructions. The CDH2 ELISA kit was purchased from Cusabio Biotech (Wuhan, China) and the ADAM10 ELISA kit was from Elabscience Biotechnology (Wuhan, China), respectively. A standard curve was created and the sample amount of CDH2 or ADAM10 was further extrapolated.

### Plasma cytokines and chemokines analyses

6 ml of whole blood samples were collected with ethylenediaminetetraacetic acid (EDTA) as anticoagulant before intake of the next dose of methadone. The plasma was obtained from the supernatant of whole blood after centrifugation at 2000×g in a Kubota 2800 centrifuge (Kubota Co., Osaka, Japan) for 20 min at 4°C, then dispensed into 1 ml/microcentrifuge tube and frozen at -80°C until use.

Cytokine IL-7 level was determined using the Milliplex^®^ MAP human cytokine kit (Millipore, Billerica, MA). All sample data were acquired from a MAGPIX^®^ System (Luminex Corp., Austin, TX).

### Statistical analyses

All analyses related to general demographic data were calculated by SAS software, version 9.4 (SAS Institute, Inc., Cary, NC, USA). Association analyses between SNPs on *CDH2* and blood pressure, plasma level of CDH2 were calculated by a general linear model (GLM). The non-normal distribution in variables of plasma CDH2, IL-7, ADAM10, and blood pressure were confirmed by the Shapiro-Wilk test. *P*-values in the GLM were calculated by permutation test with 10,000 random shuffles on the dependent variables using R software (R Development Core Team 2015). Multiple testing was adjusted by the false discovery rate using the MULTTEST procedure in the SAS software. Variable correlations of all phenotypes were explored using correlation matrix heatmap from the GAP software [32]. Pairwise correlation of variables in the heatmap was calculated by Spearman correlation coefficient. Independent variables, which had a permutation *P*-value less than 0.1 in a univariate regression, were considered to be potential predictors of dependent variables (plasma CDH2, IL-7 and ADAM10) in a multivariate regression. Independent variables in the final multivariate regression model were selected using a stepwise procedure and *P*-values of regression coefficients were calculated by permutation tests. The multicollinearity of independent variables was detected by the variance inflation factor (VIF). A VIF less than 4 indicated no multicollinearity. The covariates of medications other than methadone were adjusted by GLM and regression permutation test. All missing values were excluded from the denominator. A *P*-value <0.05 was set as an indication for statistical significance.

## Results

344 MMT patients from a cohort of 360 total recruitment had passed the genomewide genotyping quality check, and were included for analyses [31]. The average age of the 344 patients was 38 years, and 82% were males (Table 1). Average blood pressures were 125.51 and 76.69 mmHg in systolic and diastolic blood pressure respectively. Approximately 50% of the patients were poor responders, who showed a positive urine morphine test following MMT. 22.55% patients were tested positive for HIV, and among them, 95% were also infected with HCV. Three patients receiving antiretroviral therapy were included in analyses in this study. All medications other than methadone were also recorded (S1 Table).

**Table 1:** General demography of methadone maintenance treatment patients.

Variable	n	Mean ± SD	%
Age (year)	344	38.16 ± 7.69	
Gender (Male)	281		81.69
BMI (kg/m^2^)	341	23.64 ± 3.52	
Methadone dosage (mg/day)	344	55.22 ± 28.47	
Systolic blood pressure (mmHg)	331	125.51 ± 18.12	
Diastolic blood pressure (mmHg)	331	76.69 ± 12.39	
Plasma CDH2 (ng/ml)	344	16.48 ± 13.11	
Plasma ADAM10 (ng/ml)	341	26.95 ± 6.35	
Plasma IL-7 (pg/ml)	339	6.65 ± 8.25	
Urine morphine test (+)	173		50.58
HIV (+)	76		22.55
HCV (+)	313		94.56

BMI, Body Mass Index; HIV, Human immunodeficiency virus; HCV, Hepatitis C virus; (+), positive in the test; SD, standard deviation.

### Methadone maintenance therapy and study scheme

**Fig 1:**
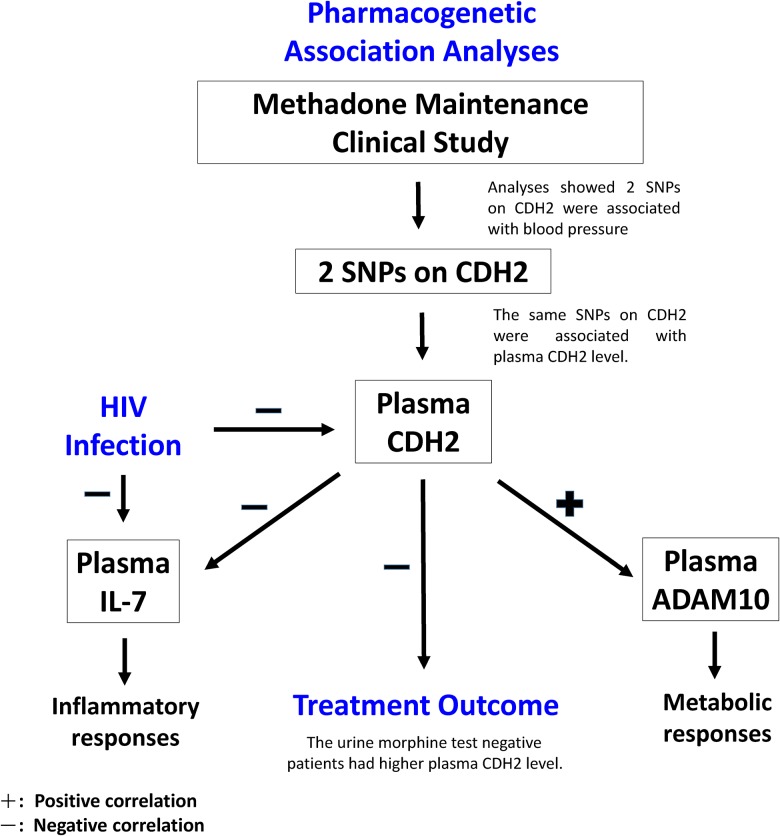
A schematic design from a pharmacogenomic study in methadone maintenance therapy. This design has led to systemic discovery of some hidden pathways among plasma IL-7, ADAM10 and CDH2, and their contributions to the methadone treatment responses.

This study has identified novel network involving methadone treatment outcome using systemic correlation analyses of plasma CDH2 (Fig 1). In pharmacogenetic association analyses between the SNPs in *CDH2* and the methadone treatment responses, two SNPs showed significant associations with blood pressure and plasma CDH2 level. Further systemic correlation analyses showed multiple significant correlations among the treatment responses. In order to clarify the hierarchy of correlations among the plasma level of CDH2 and other treatment responses, we used multiple linear regression method that selects variables step-wisely from all significant variables in the univariate regression to decipher the networks related to the plasma CDH2 level. Status of HIV infection showed a significant correlation with the plasma levels of CDH2 and cytokine IL-7. Patients with a higher plasma level of CDH2 showed a negative treatment outcome measured by the urine morphine test. Moreover, the plasma level of CDH2 showed a negative correlation with the plasma cytokine IL-7 level. Plasma level of IL-7 was mainly correlated with current corrected QT interval in the electrocardiogram (QTc) and gooseflesh skin withdrawal symptom score. As plasma CDH2 is released from the metabolism of ADAM10 [16, 17], we further analyzed the correlation between the level of ADAM10 and other treatment responses and found that plasma level of ADAM10 was significantly correlated with plasma levels of vitamin D metabolite, nicotine metabolite, and *R*-methadone. This network construction scheme indicated that methadone treatment outcome was regulated in part through the CDH2 dependent pathway.

### Association analyses of *CDH2* genetic polymorphisms

31 SNPs located within the *CDH2* genetic encoding region, well covering from intron 1 to intron 15, were selected in this study (S2 Table). Two SNPs, rs8094439 and rs17446819, at intron 2 showed significant associations with both systolic (general linear model (GLM), permutation *P* = 0.006, and 0.009, respectively) and diastolic (GLM, permutation *P* = 0.002, and 0.003, respectively) blood pressures and plasma level of CDH2 protein (GLM, permutation *P* = 0.002, and 0.003, respectively) (Table 2). The minor homozygote carriers had a higher systolic and diastolic blood pressure, and higher plasma CDH2 level than the major allele type carriers. It was mainly contributed by the urine morphine test negative patients (S3 Table).

**Table 2:** Association analyses between SNPs in *CDH2* gene and blood pressure or plasma level of CDH2.

SNP	Systolic blood pressure (mmHg)			Diastolic blood pressure (mmHg)			Plasma CDH2 level (ng/ml)		
	n	Mean ± SD	*P*-value (FDR)	n	Mean ± SD	*P*-value (FDR)	n	Mean ± SD	*P*-value (FDR)
rs8094439 (Intron 2) ^a^									
GG	227	125.98 ± 17.45	**0.006** ^b^	227	77.21 ± 11.88	**0.002** ^b^	236	15.98 ± 13.21	**0.002** ^b^
AG	92	122.72 ± 17.85	(0.06)	92	74.28 ± 12.49	**(0.031)**	95	15.61 ± 11.59	**(0.043)**
AA	11	140.55 ± 26.57	**0.022** ^c^	11	87.45 ± 15.8	**0.006** ^c^	12	31.21 ± 13.69	**0.002** ^c^
rs17446819 (Intron 2)									
AA	227	125.85 ± 17.45	**0.009** ^b^	227	77.22 ± 11.87	**0.003** ^b^	235	16 ± 13.27	**0.003** ^b^
AC	91	122.95 ± 17.81	(0.06)	91	74.46 ± 12.44	**(0.031)**	94	15.59 ± 11.65	**(0.043)**
CC	11	140.5 ± 26.57	**0.025** ^c^	11	87.45 ± 15.8	**0.007** ^c^	12	31.21 ± 13.69	**0.003** ^c^

SD, standard deviation.

^a^ Intron2 is according to the isoform of *CDH2* mRNA (NM_001792).

^b^ General linear model of permutation *P*-value.

^c^ General linear model of permutation *P*-value after adjusted for all other taken medications.

Parenthesis, False Discovery Rate (FDR). Bold font, *P* < 0.05.

### Systemic correlation analyses among all methadone treatment responses

**Fig 2:**
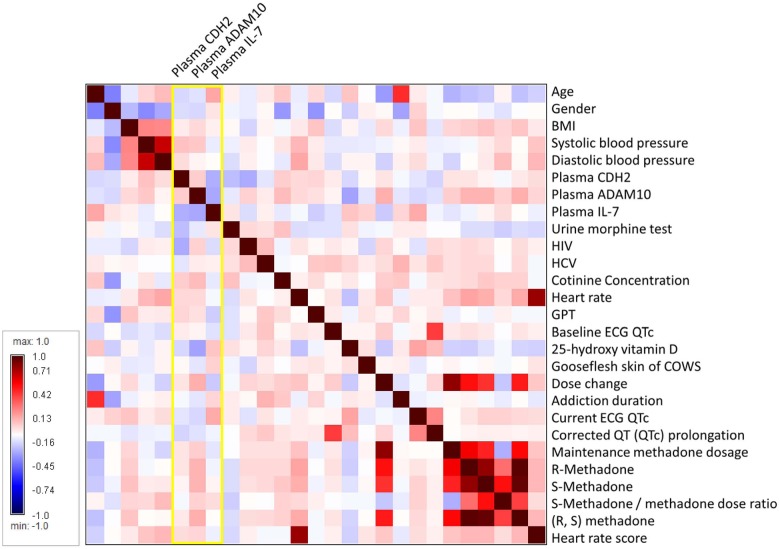
Correlation matrix heatmap of the study variables. Spearman correlation coefficients are presented by a blue-white-red color scheme. Dark red indicates a more positive correlation; dark blue indicates a more negative correlation; white indicates no correlation.

Correlation matrix showed that the plasma level of CDH2 was negatively associated with plasma level of IL-7, HIV status, and the results of urine morphine test (Fig 2, deep blue in the heatmap). Plasma level of IL-7 was negatively associated with plasma levels of CDH2 and ADAM10 (deep blue in the heatmap), and positively associated with age and the corrected current QT interval (QTc) in electrocardiogram (dark red in the heatmap). Plasma level of ADAM10 was negatively associated with plasma levels of IL-7 and 25-hydroxy vitamin D (deep blue in the heatmap), and positively associated with the levels of cotinine concentration, *R*-methadone, *S*-methadone and *(R*, *S)* methadone (dark red in the heatmap), and dose change.

Using univariate regression analyses with permutations, the plasma CDH2 level showed correlations with plasma HIV-antibody test results (*P* = 0.01), urine morphine test results, which is a surrogate for methadone treatment outcome (*P* = 0.005), and plasma level of IL-7 (*P* = 0.002) (S4 Table). The plasma IL-7 level was correlated with age (*P* = 0.0001), plasma CDH2 (*P* = 0.001), plasma level of ADAM10 (*P* = 0.008) and current ECG QTc (*P* = 0.001). The plasma level of ADAM10 was correlated with dose change (*P* = 0.0004), plasma levels of IL-7 (*P* = 0.01), *R*-Methadone (*P* = 0.0004), *S*-Methadone (*P* = 0.003), (*R*, *S*) methadone (*P* = 0.0004), cotinine concentration (*P* = 0.002) and 25-hydroxy vitamin D (*P* = 0.0004).

### Plasma CDH2 level is correlated with status of HIV infection, plasma level of cytokine IL-7, and treatment outcome

In multivariate regression analyses, plasma CDH2 level was significantly associated with plasma level of cytokine IL-7 (β = -0.32, *P* = 0.0008), HIV infection status (β = -5.35, *P* = 0.003), and the urine morphine test results (β = -3.24, *P* = 0.028) (Table 3). In further subgrouping analyses, the HIV positive MMT patients had a lower plasma CDH2 level than the HIV negative patients (GLM, permutation *P* = 0.009) (Table 4). Subjects with a higher plasma CDH2 level showed a better treatment outcome (GLM, permutation *P* = 0.006).

**Table 3:** Multivariate regression analyses of the plasma CDH2 level (ng/ml).

Variable ^a^	β	S.E.	*P*-value ^b^	Adjusted ^c^	Partial r^2^	VIF
Plasma IL-7 (pg/ml)	-0.32	0.09	**0.0008**	**0.0009**	0.036	1.03
HIV (+ / -)	-5.35	1.73	**0.003**	**0.002**	0.032	1.04
Urine morphine (+ / -)	-3.24	1.47	**0.028**	**0.039**	0.012	1.04
Cotinine Concentration (ng/ml)	0.01	0.004	0.06	0.07	0.012	1.03
Heart rate (beats/min)	0.11	0.06	0.07	0.06	0.013	1.05
Systolic blood pressure (mmHg)	0.05	0.04	0.2	0.29	0.005	1.04

^a^ n = 319, F = 6.38, *P*<0.0001, adjusted r^2^ = 9.24%.

^b^ Permutation *P*-value.

^c^ Permutation *P*-value adjusted for all other taken medications.

Bold font, *P* < 0.05. HIV, human immunodeficiency virus; β, stepwise regression coefficient; S.E., standard error of regression coefficient; VIF, variance inflation factor.

**Table 4:** Plasma level of CDH2 and IL-7 were associated with HIV infection status and the urine morphine test outcome.

Variable	Plasma CDH2 level (ng/ml)			Plasma IL-7 level (pg/ml)		
	n	Mean ± SD	*P*-value	n	Mean ± SD	*P*-value
**HIV**			**0.009** ^a^			**0.013** ^a^
HIV (+)	76	12.97 ± 10.79	**0.008** ^b^	76	4.59 ± 5.77	**0.045** ^b^
HIV (-)	261	17.41 ± 13.64		257	7.31 ± 8.83	
**Urine morphine test**			**0.006** ^a^			0.31 ^a^
Morphine (+)	173	14.59 ± 10.82	**0.005** ^b^	170	7.14 ± 8.69	0.38 ^b^
Morphine (-)	169	18.49 ± 14.92		167	6.23 ± 7.80	

^a^ General linear model of permutation *P*-value.

^b^ Permutation *P*-value adjusted for all other taken medications.

Bold font, *P* < 0.05. HIV, human immunodeficiency virus; SD, standard deviation.

### Plasma level of IL-7 is correlated with QT interval in electrocardiogram and gooseflesh skin withdrawal symptom

Status of HIV infection was correlated with plasma level of IL-7 (Table 4). HIV positive MMT patients had a lower plasma level of IL-7 than HIV negative patients (GLM, permutation *P* = 0.013). Plasma level of IL-7 was associated with corrected current QT interval (QTc) in electrocardiogram (β = 0.08, *P* = 0.0001), gooseflesh skin withdrawal symptom score (β = 3.92, *P* = 0.007), and plasma CDH2 level (β = -0.12, *P* = 0.003) using multivariate regression analyses after the permutation analyses (S5 Table).

### Plasma level of ADAM10 is correlated with the levels of metabolites of vitamin D, methadone, and nicotine

Plasma level of CDH2 is affected by the activities of matrix metalloproteinase (MMP), which catalyzes the release of extracellular domain of CDH2 circulating in the blood stream. Correlation matrix heatmap showed that plasma level of IL-7 was negatively associated with plasma levels of CDH2 and ADAM10 (Fig 2, deep blue in the heatmap) and positively associated with age and the current QTc in ECG (Fig 2, deep red in the heatmap). In order to decipher the mechanism underlying the increase in plasma CDH2 level, we also measured the plasma ADAM10 concentration. A multivariate regression analysis showed that plasma level of ADAM10 was associated with the plasma levels of 25-hydroxy vitamin D (β = -0.04, *P* = 0.002), nicotine metabolite cotinine (β = 0.01, *P* = 0.006), and *R*-methadone (β = 0.01, *P* = 0.004) (S6 Table).

### A network was constructed among plasma CDH2, IL-7 and ADAM10

In summary, plasma CDH2 level may be influenced by status HIV of infection. It may also play a role in predicting the effectiveness of methadone maintenance treatment (*P* = 0.039 in multivariate regression, *P* = 0.006 in association analyses) (S1 Fig). Using systemic GAP relation analyses, multiple methadone treatment responses showed correlations with plasma CDH2 level. The multivariate regression analyses demonstrated a clearer relation rank of order and constructed the relations among plasma levels of CDH2, cytokine IL-7, and CDH2 metabolizing MMP, ADAM10. The cytokine IL-7 network is correlated with QTc interval and the withdrawal symptom gooseflesh skin. The ADAM10 network is correlated with the metabolites of vitamin D, the metabolite of nicotine cotinine, and the plasma *R*-methadone.

## Discussion

In this study, we performed genetic association analyses between SNPs in the CDH2 genetic region from a genomewide database and the methadone treatment responses. Two SNPs, rs8094439 and rs17446819, located at intron 2 of *CDH2* showed significant associations with systolic and diastolic blood pressure. The two SNPs also showed significant associations with plasma level of CDH2. This result suggested equilibrium between the translation of CDH2 and MMP activity, which regulates the plasma level of CDH2. In further multiple regression analyses, plasma CDH2 level showed correlations with level of cytokine IL-7, HIV infection status, and the urine morphine test outcome. In this cohort, the HIV infection status was measured by plasma HIV antibody only. No information regarding viral load is available. Nevertheless, the results in this study remain the same when the data from the three patients who received antiretroviral therapy were removed from analysis. Details of HIV infection status vs. the plasma levels of CDH2 or IL-7 warrant further investigation. The results provided new information with regard to the environmental effects contributed by the status of HIV infection, and led to the discovery of a novel systemic regulatory mechanism involving CDH2 and the cytokine IL-7 in the methadone treatment outcome in heroin dependent patients.

For the two SNPs at intron 2 in *CDH2* genetic coding region that showed significant associations with blood pressure in MMT patients, the minor genotype carriers showed a higher plasma CDH2 level and higher blood pressure than the major allele type carriers, particularly in patients with negative results from the urine morphine test (S3 Table). This is a first study demonstrating that *N*-cadherin (CDH2) was detectable from the blood plasma of methadone maintenance patients. In univariate correlation analyses, the plasma CDH2 level was marginally correlated with systolic blood pressure. The higher the plasma CDH2 level, the higher was the systolic blood pressure. Using multiple regression analyses, which may be able to remove the low correlation factors, plasma level of CDH2 was significantly correlated with plasma IL-7 level, HIV infection status, and the urine morphine test results in a rank order (Table 3). The strong negative correlation between plasma levels of CDH2 and IL-7 indicated a novel interaction network in systemic pharmacological status of MMT patients. The negative correlation between the plasma level of CDH2 and HIV infection status further suggested that HIV infection may have an impact on decrease in plasma CDH2 level. Moreover, the treatment outcome evaluated by the urine morphine test results also showed a negative correlation with the plasma CDH2 level. The higher the plasma CDH2 concentration, the better treatment outcome was observed in the urine morphine test negative patients.

In order to further clarify the mechanisms underlying the regulation of plasma levels of CDH2 and cytokine IL-7, we measured the levels of MMP, ADAM10, which is one of the key enzymes that determine the plasma level of CDH2 [16, 17]. We found that the plasma ADAM10 level showed a positive correlation with the severity of cigarette smoking measured by the plasma level of a nicotine metabolite, cotinine, and the plasma *R*-methadone concentration (S6 Table). Other factors related to plasma ADAM10 concentration was the level of metabolite of vitamin D, 25-hydroxy vitamin D, yet it was negatively correlated. Since the plasma CDH2 level is regulated by the enzymatic activity of MMP ADAM10 [16, 17] and is correlated with systolic blood pressure, the correlation explained in part the reason why an MMP inhibitor may decrease blood pressure [33]. Although the mechanism between the relation of plasma CDH2 and blood pressure is not fully understood, CDH2 has been reported involving the heart integrity [34] and arteriolar myogenic reactivity [11]. This implicated that the genetic polymorphisms in *CDH2* may be an indicator for subgroups of patients regarding the difference in blood pressure in MMT patients.

Genomic studies on heroin dependence in other ethnic groups are so far focusing on candidate gene association analyses [35]. The present study is the first published study analyzing the plasma protein identified from the results of the CDH2 genomic association analyses. A plasma protein network regarding the influence of HIV infection on treatment outcome of MMT was discovered. We identified two plasma proteins, CDH2 and IL-7, that were influenced by the HIV serological infection status. These two proteins could potentially indicate HIV infection clinically. Moreover, the plasma CDH2 may also indicate the treatment outcome of MMT evaluated by urine morphine test results (Table 4). The higher level of plasma CDH2, the greater chance that patients would be tested negative in the urine morphine, i.e., remaining abstinent from opioid use. Plasma ADAM10 level was mostly correlated with the metabolites and less influenced by the plasma CDH2 (S4 Table).

In conclusion, the present study identified associations among the status of HIV, methadone maintenance treatment outcome measured by urine morphine test results, and plasma CDH2 level. Medications taken other than methadone or self reported other substance use did not affect the results of the analyses in the present study. However, we think the study warrants further replication in other population or ethnic groups. This study was not meant to identify causality. Future investigation using animal or cellular studies would provide further information. Nevertheless, the present study has demonstrated a potentially effective strategy to identify the network involving methadone treatment outcome using systemic correlation analyses.

## Supporting information

S1 Fig Summary of the significant network among plasma levels of IL-7, ADAM10 and CDH2 after adjusted for all other taken medications. (DOC)

S1 Table Numbers of MMT patients who also took other medications. (DOC)

S2 Table Single nucleotide polymorphisms analyzed in *CDH2*. (DOC)

S3 Table Association analysis between SNPs in *CDH2* gene and blood pressure or plasma level of CDH2 in the urine morphine test negative patients. (DOC)

S4 Table Univariate regression analyses between the plasma levels of CDH2, IL-7 or ADAM10 and methadone treatment responses. (DOC)

S5 Table Multivariate regression analyses of the plasma IL-7 level (pg/ml). (DOC)

S6 Table Multivariate regression analyses of the plasma ADAM10 level (ng/ml). (DOC)
