# Supplementary material for: Pharmacogenomics study on cadherin 2 network with regard to HIV infection and methadone treatment outcome
Source: PLoS One. 2017 Mar 30;12(3):e0174647. doi: 10.1371/journal.pone.0174647 (PMC5373543; doi:10.1371/journal.pone.0174647)

**S1 Fig.** **Summary of the significant network among plasma levels of IL-7, ADAM10 and CDH2 after adjusted for all** **other taken medications.** Multiple regression analysis showed IL-7 and CDH2 were strongly correlated, and they may be involved with the MMT treatment outcomes, HIV infection and aging. Plasma ADAM10 level is significantly correlated with plasma concentrations of *R*-methadone, 25-hydroxy vitamin D and nicotine metabolite cotinine.


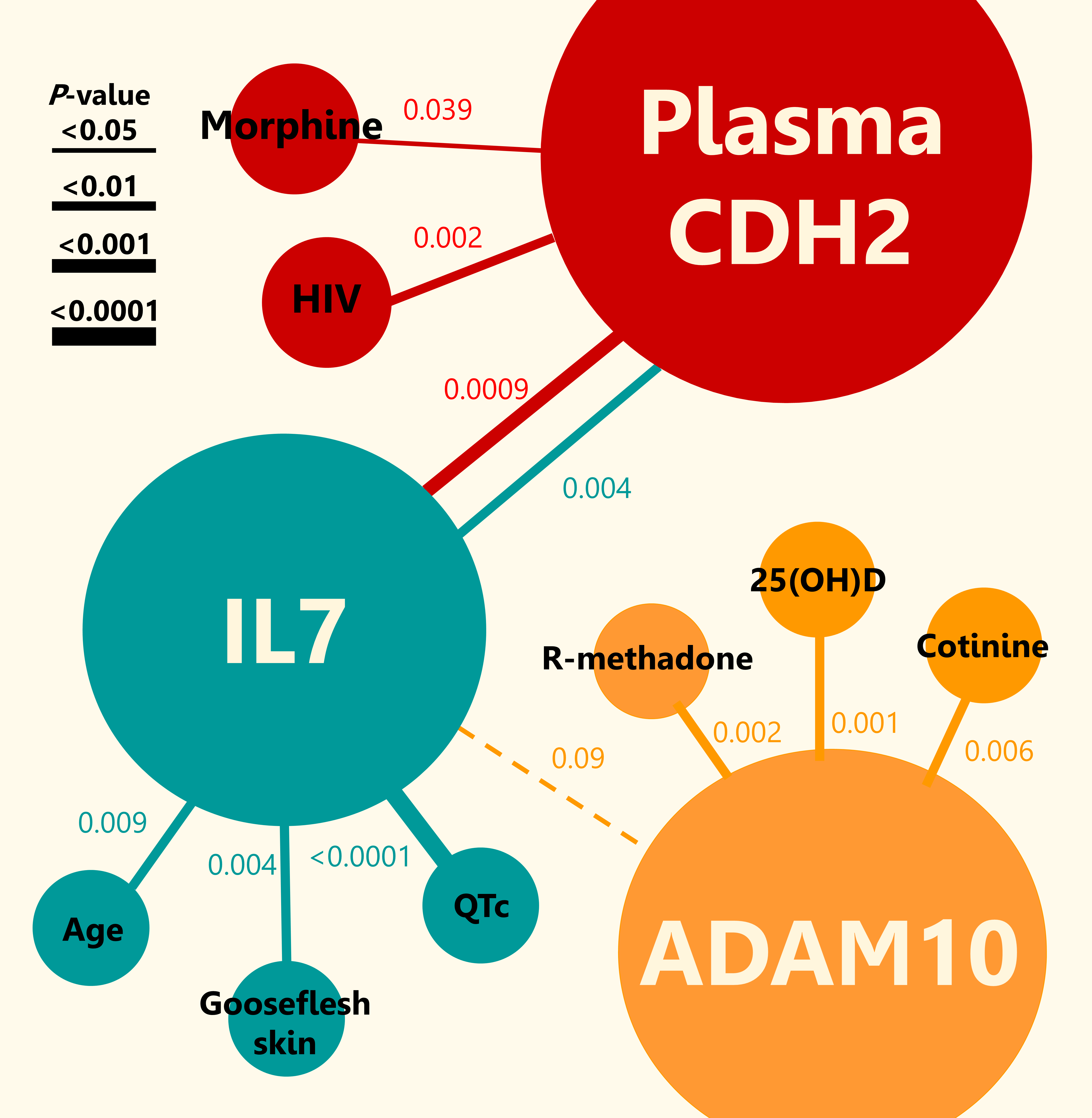

Supplement: S1 Fig — (DOC) [file pone.0174647.s001.doc]
