# Supplementary material for: Pharmacogenomics study on cadherin 2 network with regard to HIV infection and methadone treatment outcome
Source: PLoS One. 2017 Mar 30;12(3):e0174647. doi: 10.1371/journal.pone.0174647 (PMC5373543; doi:10.1371/journal.pone.0174647)
Supplement: S1 Table — (DOC) [file pone.0174647.s002.doc]

| S1 Table. Numbers of MMT patients who also took other medications and substances. | | |
| --- | --- | --- |
| Variable | n | % |
| Medications from medical records |  |  |
| Antipsychotics | 23 | 6.69 |
| Sedative-hypnotics | 120 | 34.88 |
| Anti-AIDS | 3 | 0.87 |
| Hepatitis medicine | 15 | 4.36 |
| Diabetes | 5 | 1.45 |
| Antihypertensives | 6 | 1.74 |
| Traditional Chinese medicine | 13 | 3.78 |
| Other substances from self-report |  |  |
| Opiates | 217 | 63.08 |
| Alcohol | 112 | 32.56 |
| Betel nut | 90 | 26.16 |
| Amphetamine | 62 | 18.02 |
| Crack | 0 | 0.00 |
| Cocaine | 0 | 0.00 |
| Cannabis | 0 | 0.00 |
