# Supplementary material for: Pharmacogenomics study on cadherin 2 network with regard to HIV infection and methadone treatment outcome
Source: PLoS One. 2017 Mar 30;12(3):e0174647. doi: 10.1371/journal.pone.0174647 (PMC5373543; doi:10.1371/journal.pone.0174647)
Supplement: S2 Table — (DOC) [file pone.0174647.s003.doc]

S2 Table. Single nucleotide polymorphisms analyzed in *CDH2.*

| SNP | Chr.Position | Location | Allele | MAF | HWP | Call rate |
| --- | --- | --- | --- | --- | --- | --- |
| rs9945664 | 25533472 | Intron 15 | T/C | 0.065 | 0.86 | 100.00% |
| rs4363925 | 25534039 | Intron 15 | G/A | 0.43 | 0.19 | 100.00% |
| rs11083239 | 25537685 | Intron 15 | T/A | 0.469 | 0.86 | 98.80% |
| rs2847361 | 25541288 | Intron 15 | G/T | 0.413 | 0.82 | 100.00% |
| rs1041985 | 25543387 | Exon 15 | G/A | 0.398 | 0.17 | 100.00% |
| rs11083241 | 25551961 | Intron 14 | A/G | 0.443 | 0.27 | 100.00% |
| rs17445700 | 25553075 | Intron 14 | G/A | 0.432 | 1.00 | 100.00% |
| rs17445707 | 25553342 | Intron 14 | G/T | 0.438 | 0.86 | 100.00% |
| rs17445714 | 25553426 | Intron 14 | T/C | 0.039 | 0.82 | 100.00% |
| rs1443454 | 25554315 | Intron 14 | G/A | 0.129 | 0.96 | 100.00% |
| rs17468095 | 25555446 | Intron 14 | C/T | 0.045 | 1.00 | 100.00% |
| rs1220156 | 25556552 | Intron 14 | T/C | 0.411 | 0.94 | 99.70% |
| rs17445742 | 25557305 | Intron 14 | G/T | 0.045 | 1.00 | 100.00% |
| rs8092870 | 25562831 | Intron 14 | C/T | 0.177 | 0.66 | 100.00% |
| rs10502506 | 25576002 | Intron 7 | C/T | 0.302 | 0.42 | 100.00% |
| rs17445819 | 25591325 | Intron 4 | T/C | 0.047 | 0.32 | 100.00% |
| rs539075 | 25613439 | Intron 2 | T/G | 0.324 | 0.51 | 99.70% |
| rs11564412 | 25619000 | Intron 2 | C/T | 0.275 | 0.86 | 100.00% |
| rs500643 | 25629299 | Intron 2 | G/T | 0.334 | 0.77 | 100.00% |
| rs1220032 | 25629595 | Intron 2 | T/G | 0.195 | 0.38 | 100.00% |
| rs17446134 | 25633996 | Intron 2 | G/A | 0.047 | 0.32 | 100.00% |
| rs17468531 | 25638608 | Intron 2 | A/G | 0.328 | 1.00 | 100.00% |
| rs512362 | 25647288 | Intron 2 | A/G | 0.471 | 0.61 | 100.00% |
| rs894679 | 25658907 | Intron 2 | C/T | 0.286 | 0.91 | 100.00% |
| rs528438 | 25673875 | Intron 2 | C/T | 0.167 | 0.99 | 100.00% |
| rs1785835 | 25684095 | Intron 2 | A/G | 0.45 | 0.32 | 98.00% |
| rs1220034 | 25699351 | Intron 2 | G/A | 0.315 | 0.51 | 99.70% |
| rs8094439 | 25712607 | Intron 2 | G/A | 0.173 | 0.62 | 99.70% |
| rs17446819 | 25716889 | Intron 2 | A/C | 0.173 | 0.59 | 99.10% |
| rs10513896 | 25731520 | Intron 1 | C/T | 0.346 | 0.91 | 100.00% |
| rs1941384 | 25734936 | Intron 1 | G/A | 0.291 | 0.91 | 100.00% |
| Localization, according to the isoform of *CDH2* mRNA(NM_001792). | | | | | | |
| MAF, Minor allele frequency. HWP, *P*-value of Hardy-Weinberg equilibrium test. | | | | | | |
