# Supplementary material for: Pharmacogenomics study on cadherin 2 network with regard to HIV infection and methadone treatment outcome
Source: PLoS One. 2017 Mar 30;12(3):e0174647. doi: 10.1371/journal.pone.0174647 (PMC5373543; doi:10.1371/journal.pone.0174647)
Supplement: S3 Table — (DOC) [file pone.0174647.s004.doc]

S3 Table. Association analysis between SNPs in *CDH2* gene and blood pressure or plasma level of CDH2 in the urine morphine test **negative** patients.

|  | Systolic blood pressure (mmHg) | | | | |  | Diastolic blood pressure (mmHg) | | | | |  | Plasma CDH2 level (ng/ml) | | | | |
| --- | --- | --- | --- | --- | --- | --- | --- | --- | --- | --- | --- | --- | --- | --- | --- | --- | --- |
| SNP | n | Mean | ± | SD | *P*-value |  | n | Mean | ± | SD | *P*-value |  | n | Mean | ± | SD | *P*-value |
|  |  |  |  |  | (FDR) |  |  |  |  |  | (FDR) |  |  |  |  |  | (FDR) |
| rs8094439 (Intron 2)^a^ | |  |  |  |  |  |  |  |  |  |  |  |  |  |  |  |  |
| GG | 114 | 127.84 | ± | 17.25 | **0.001** ^b^ |  | 114 | 78.75 | ± | 11.71 | **0.001** ^b^ |  | 117 | 17.72 | ± | 15.52 | **0.022** ^b^ |
| AG | 43 | 117.51 | ± | 14.05 | **( 0.019 )** |  | 43 | 72.33 | ± | 11.20 | **( 0.022 )** |  | 44 | 17.51 | ± | 11.66 | ( 0.26 ) |
| AA | 7 | 137.57 | ± | 21.00 |  |  | 7 | 86.71 | ± | 11.90 |  |  | 7 | 34.52 | ± | 14.69 |  |
| rs17446819 (Intron 2) | |  |  |  |  |  |  |  |  |  |  |  |  |  |  |  |  |
| AA | 114 | 127.59 | ± | 17.29 | **0.002** ^b^ |  | 114 | 78.76 | ± | 11.68 | **0.002** ^b^ |  | 116 | 17.78 | ± | 15.63 | **0.024** ^b^ |
| AC | 42 | 117.88 | ± | 14.01 | **( 0.023 )** |  | 42 | 72.67 | ± | 11.10 | **( 0.033 )** |  | 43 | 17.51 | ± | 11.79 | ( 0.26 ) |
| CC | 7 | 137.57 | ± | 21.00 |  |  | 7 | 86.71 | ± | 11.90 |  |  | 7 | 34.52 | ± | 14.69 |  |

SD, standard deviation.

^a^ Intron2 is according to the isoform of CDH2 mRNA (NM_001792).

^b^ General linear model of permutation *P*-value.

Parenthesis, False Discovery Rate (FDR). Bold font, *P* < 0.05.
