# Supplementary material for: Pharmacogenomics study on cadherin 2 network with regard to HIV infection and methadone treatment outcome
Source: PLoS One. 2017 Mar 30;12(3):e0174647. doi: 10.1371/journal.pone.0174647 (PMC5373543; doi:10.1371/journal.pone.0174647)
Supplement: S4 Table — (DOC) [file pone.0174647.s005.doc]

S4 Table. Univariate regression analyses between the plasma levels of CDH2, IL-7 or ADAM10 and methadone treatment responses.

| Variable | n | β | *P*-value | Adjusted |
| --- | --- | --- | --- | --- |
| **Plasma CDH2 (ng/ml)** |  |  |  |  |
| **General** |  |  |  |  |
| Age | 344 | -0.18 | **0.043** | **0.038** |
| Cotinine concentration (ng/ml) | 344 | 0.007 | 0.07 | 0.08 |
| ALT (U/L) | 332 | 0.002 | 0.84 | 0.80 |
| Plasma ADAM10 (ng/ml) | 341 | 0.20 | 0.07 | 0.09 |
| HIV (+/-) | 337 | -4.44 | **0.010** | **0.009** |
| Urine morphine test(+/-) | 342 | -3.90 | **0.005** | **0.004** |
| **Cardiovascular** |  |  |  |  |
| Systolic blood pressure (mmHg) | 331 | 0.08 | 0.05 | 0.08 |
| Diastolic blood pressure (mmHg) | 331 | 0.05 | 0.44 | 0.53 |
| Baseline ECG QTc (ms) | 222 | 0.07 | **0.048** | 0.06 |
| Heart rate (beats/min) | 343 | 0.12 | **0.047** | **0.038** |
| **Growth factor** |  |  |  |  |
| Plasma IL-7 (pg/ml) | 339 | -0.30 | **0.002** | **0.002** |
| **Plasma IL-7 (pg/ml)** |  |  |  |  |
| **General** |  |  |  |  |
| Age | 339 | 0.24 | **0.0001** | **0.0002** |
| 25-hydroxy vitamin D (nM) | 339 | 0.03 | 0.06 | 0.06 |
| Gooseflesh skin of COWS | 339 | 2.80 | **0.015** | **0.013** |
| Dose change (Tolerance) | 328 | -0.04 | **0.016** | **0.035** |
| Plasma CDH2 (ng/ml) | 339 | -0.12 | **0.001** | **0.003** |
| Plasma ADAM10 (ng/ml) | 337 | -0.19 | **0.008** | **0.009** |
| Addiction duration (year) | 339 | 0.14 | **0.023** | **0.017** |
| ALT (U/L) | 328 | -0.008 | 0.19 | 0.18 |
| HIV (+/-) | 333 | -2.72 | **0.013** | **0.049** |
| Urine morphine test (+/-) | 337 | 0.92 | 0.31 | 0.38 |
| **Cardiovascular** |  |  |  |  |
| Baseline ECG QTc (ms) | 219 | 0.03 | 0.15 | 0.73 |
| Current ECG QTc (ms) | 283 | 0.07 | **0.001** | **0.0003** |
| Corrected QT (QTc) prolongation | 174 | -0.01 | 0.55 | 0.24 |
| **Plasma ADAM10 (ng/ml)** |  |  |  |  |
| **General** |  |  |  |  |
| Maintenance methadone dosage (mg/day) | 341 | 0.026 | **0.028** | **0.019** |
| Dose change (Tolerance) | 330 | 0.039 | **0.0004** | **0.0003** |
| Plasma CDH2 (ng/ml) | 341 | 0.047 | 0.07 | 0.08 |
| Plasma IL-7 (pg/ml) | 337 | -0.11 | **0.010** | **0.010** |
| **Plasma concentrations of methadone and its metabolites** | | | | |
| *R*-Methadone (ng/ml) | 341 | 0.01 | **0.0004** | **0.0002** |
| *S*-Methadone (ng/ml) | 341 | 0.01 | **0.003** | **0.002** |
| *S*-Methadone / methadone dose ratio | 341 | 0.49 | **0.029** | **0.030** |
| (*R, S*) methadone (ng/ml) | 341 | 0.006 | **0.0004** | **0.0005** |
| Cotinine concentration (ng/ml) | 341 | 0.006 | **0.002** | **0.001** |
| 25-hydroxy vitamin D (nM) | 341 | -0.045 | **0.0004** | **0.0003** |
| **Cardiovascular** |  |  |  |  |
| Systolic blood pressure (mmHg) | 328 | 0.036 | 0.06 | 0.054 |
| Heart rate score | 340 | 1.24 | **0.040** | **0.045** |

HIV, Human immunodeficiency virus.  β, regression coefficient. *P*-value, permutation *P*-value.

Adjusted, permutation *P*-value adjusted for all other taken medications. Bold font, *P* < 0.05
