# Supplementary material for: Pharmacogenomics study on cadherin 2 network with regard to HIV infection and methadone treatment outcome
Source: PLoS One. 2017 Mar 30;12(3):e0174647. doi: 10.1371/journal.pone.0174647 (PMC5373543; doi:10.1371/journal.pone.0174647)
Supplement: S5 Table — (DOC) [file pone.0174647.s006.doc]

S5 Table. Multivariate regression analyses of the plasma IL-7 level (pg/ml).

| Variable | β | S.E. | *P*-value | Adjusted | Partial r^2^ | VIF |
| --- | --- | --- | --- | --- | --- | --- |
| Current ECG QTc (ms) | 0.08 | 0.02 | **0.0001** | **<0.0001** | 0.045 | 1.04 |
| Gooseflesh skin of COWS | 3.92 | 1.19 | **0.007** | **0.004** | 0.042 | 1.02 |
| Plasma CDH2 (ng/ml) | -0.12 | 0.04 | **0.003** | **0.004** | 0.036 | 1.03 |
| Age | 0.16 | 0.06 | **0.015** | **0.009** | 0.032 | 1.08 |
| Dose change (Tolerance) | -0.03 | 0.02 | 0.1 | 0.13 | 0.012 | 1.09 |
| HIV (+/-) | -2.38 | 1.19 | **0.048** | 0.15 | 0.012 | 1.06 |

n=269, F=9.47, *P*<0.0001, adjusted r^2^=15.95%. Bold font, *P* < 0.05

HIV, human immunodeficiency virus.

β, stepwise regression coefficient. S.E., standard error of regression coefficient.

*P*-value, permutation *P*-value. VIF, variance inflation factor.

Adjusted, permutation *P*-value adjusted for all other taken medications.
