# Supplementary material for: Pharmacogenomics study on cadherin 2 network with regard to HIV infection and methadone treatment outcome
Source: PLoS One. 2017 Mar 30;12(3):e0174647. doi: 10.1371/journal.pone.0174647 (PMC5373543; doi:10.1371/journal.pone.0174647)
Supplement: S6 Table — (DOC) [file pone.0174647.s007.doc]

S6 Table. Multivariate regression analyses of the plasma ADAM10 level (ng/ml).

| Variable | β | S.E. | *P*-value | Adjusted | Partial r^2^ | VIF |
| --- | --- | --- | --- | --- | --- | --- |
| 25-hydroxy vitamin D (nM) | -0.04 | 0.012 | **0.002** | **0.001** | 0.047 | 1.06 |
| Cotinine Concentration (ng/ml) | 0.01 | 0.002 | **0.006** | **0.006** | 0.028 | 1.02 |
| *R*-Methadone (ng/ml) | 0.01 | 0.003 | **0.004** | **0.002** | 0.025 | 1.03 |
| Plasma IL-7 (pg/ml) | -0.07 | 0.041 | 0.08 | 0.09 | 0.011 | 1.05 |
| Systolic blood pressure (mmHg) | 0.02 | 0.019 | 0.22 | 0.27 | 0.005 | 1.02 |
| Plasma CDH2 (ng/ml) | 0.02 | 0.026 | 0.49 | 0.47 | 0.001 | 1.07 |

n=325, F=6.97, *P*<0.0001, adjusted r^2^=9.99%. Bold font, *P* < 0.05

β, stepwise regression coefficient. S.E., standard error of regression coefficient.

*P*-value, permutation *P*-value. VIF, variance inflation factor.

Adjusted, permutation *P*-value adjusted for all other taken medications.
